# Coordinated Upregulation of Mitochondrial Biogenesis and Autophagy in Breast Cancer Cells: The Role of Dynamin Related Protein-1 and Implication for Breast Cancer Treatment

**DOI:** 10.1155/2016/4085727

**Published:** 2016-09-26

**Authors:** Peng Zou, Longhua Liu, Louise D. Zheng, Kyle K. Payne, Masoud H. Manjili, Michael O. Idowu, Jinfeng Zhang, Eva M. Schmelz, Zhiyong Cheng

**Affiliations:** ^1^Department of Human Nutrition, Foods, and Exercise, Fralin Life Science Institute, College of Agriculture and Life Sciences, Virginia Tech, Blacksburg, VA 24061, USA; ^2^Department of Microbiology and Immunology, Massey Cancer Center, Virginia Commonwealth University, Richmond, VA 23298, USA; ^3^Department of Pathology, School of Medicine, Virginia Commonwealth University, Richmond, VA 23298, USA; ^4^Department of Statistics, Florida State University, Tallahassee, FL 32306, USA

## Abstract

Overactive mitochondrial fission was shown to promote cell transformation and tumor growth. It remains elusive how mitochondrial quality is regulated in such conditions. Here, we show that upregulation of mitochondrial fission protein, dynamin related protein-1 (Drp1), was accompanied with increased mitochondrial biogenesis markers (PGC1*α*, NRF1, and Tfam) in breast cancer cells. However, mitochondrial number was reduced, which was associated with lower mitochondrial oxidative capacity in breast cancer cells. This contrast might be owing to enhanced mitochondrial turnover through autophagy, because an increased population of autophagic vacuoles engulfing mitochondria was observed in the cancer cells. Consistently, BNIP3 (a mitochondrial autophagy marker) and autophagic flux were significantly upregulated, indicative of augmented mitochondrial autophagy (mitophagy). The upregulation of Drp1 and BNIP3 was also observed in vivo (human breast carcinomas). Importantly, inhibition of Drp1 significantly suppressed mitochondrial autophagy, metabolic reprogramming, and cancer cell viability. Together, this study reveals coordinated increase of mitochondrial biogenesis and mitophagy in which Drp1 plays a central role regulating breast cancer cell metabolism and survival. Given the emerging evidence of PGC1*α* contributing to tumor growth, it will be of critical importance to target both mitochondrial biogenesis and mitophagy for effective cancer therapeutics.

## 1. Introduction

Metabolic and nutrient homeostasis is critical for cellular function and human health. Mitochondrial alterations have been implicated in a variety of human metabolic diseases including cancer [1–3]. A hallmark of cancer metabolism is the switch from oxidative phosphorylation (OXPHOS) to robust glycolysis, and deficits of OXPHOS have been associated with malignancy and cancer cell growth [4–6]. As such, increasing efforts have been made to explore cancer therapeutics by targeting mitochondria and the metabolic switch [4, 5, 7, 8].

As the organelles where OXPHOS takes place, mitochondria are under tight content and quality control via the triad of de novo mitochondrial biogenesis, mitochondrial dynamics (fusion and fission), and mitochondrial autophagy (mitophagy) [9, 10]. Frequent fusion and fission facilitate the exchange of proteins, mtDNA, and metabolites to maintain mitochondrial integrity [9–12]. Dysregulation of the dynamic processes impairs mitochondrial function and has been reported in cancer, diabetes, and neurodegenerative diseases [9, 10, 13]. For instance, mitochondrial fission is upregulated due to Drp1 activation or overexpression in different types of tumors or cancers [14–18]. In breast cancer, activation of Drp1 promotes mitochondrial fragmentation and facilitates cancer cell migration and invasion; by contrast, ablation of Drp1 in breast cancer cells leads to mitochondria elongation and dampens their metastatic ability [14]. However, important questions remain as to how Drp1 dysregulation may interact with mitochondrial biogenesis and mitophagy and how the interactions affect mitochondrial quality control and are related to metabolic reprograming in breast cancer cells.

In this study, we found that upregulation of Drp1 was associated with reduced mitochondrial oxidative capacity in breast cancer cells. Surprisingly, mitochondrial content or number was reduced despite elevated regulators that promote mitochondrial biogenesis (i.e., PGC1*α*, NRF1, and Tfam) in breast cancer cells. These changes may arise from augmented mitophagy that removes mitochondria from cancer cells. Importantly, inhibition of Drp1 attenuated mitophagy and reversed the metabolic reprogramming, which reduced breast cancer cell viability. Our data demonstrate for the first time that the Drp1-mitophagy axis plays a key role in mitochondrial turnover and metabolic reprogramming for cell survival in breast cancer.

## 2. Materials and Methods

### 2.1. Cell Culture

Human breast cancer cells MDA-MB-231 were cultured in RPMI 1640 medium (Corning) supplemented with 10% FBS (fetal bovine serum, GeneMate), 100 units/mL penicillin, and 100 *μ*g/mL streptomycin (Hyclone) [19]. MCF-10A cells were cultured in DMEM/Ham's F-12 (1 : 1 mixture) (Caisson Labs), supplemented with 5% horse serum (Gibco), 100 units/mL penicillin and 100 *μ*g/mL streptomycin, 10 *μ*g/mL human recombinant insulin (Sigma-Aldrich), 0.02 *μ*g/mL rH-EGF (Shenandoah), 0.5 *μ*g/mL hydrocortisone (Sigma-Aldrich), and 0.1 *μ*g/mL cholera toxin (List Biological Laboratories) [20]. Cultures were seeded at a density of 2–6 × 10^4^ cells/cm^2^ and incubated at 37°C in a humidified atmosphere of 5% CO_2_, and the media were replaced every two days. For the treatment with Mdivi-1, the regular media for MDA-MB-231 cell culture were changed on the next day to fresh medium with Mdivi-1 (50–250 *μ*M, Cayman Chemical) [21–24] or vehicle (0.1% DMSO) and maintained for 24 hours before further analyses were performed. Cell viability was determined by trypan blue dye exclusion assay as described previously [25].

### 2.2. Electron Microscopy

The electron microscopy was performed as described previously [13, 26]. Briefly, the cells were trypsinized at 80%–90% confluence, washed with PBS, and then fixed in the mix of glutaraldehyde (2.5%), formaldehyde (4.4%), and picric acid (2.75%) in 0.05% sodium cacodylate buffer at 4°C overnight. Cells were rinsed in cacodylate buffer twice and incubated for 1 h at 4°C in 1% (v/v) OsO4/1% (w/v) K_4_Fe(CN)_6_ in cacodylate buffer at pH 7.4, followed by rinsing in cacodylate buffer and then in distilled water. The cells were stained with 1% (w/v) aqueous uranyl acetate for 2 h at 4°C, washed with distilled water, and dehydrated through increased graded ethanol series and embedded. Ultrathin sections were stained with lead citrate and underwent imaging analysis with electron microscopy [13].

### 2.3. Autophagy Flux Assay

MCF-10A and MDA-MB-231 cells were cultured in the media as described above and then treated with autolysosome inhibitors bafilomycin A1 (0.1 *μ*M) and leupeptin (10 *μ*g/mL) for 4 hr [27–30]. The cell lysates were prepared as previously described [31, 32], and LC3-II was detected by western blotting and image analysis to assess autophagy flux (i.e., the difference of LC3-II levels at 4 hr and at 0 hr) [27–30].

### 2.4. Mitochondrial Oxidative Capacity

Mitochondrial oxidative capacity was measured with Oxygen Consumption Rate Assay Kits (Cayman Chemical), and parameters including oxygen consumption rate (OCR), mitochondrial ATP turnover, respiration control ratio, coupling efficiency (CE), max respiration rate, and spare respiration capacity (SRC) were analyzed as described previously [33].

### 2.5. Measurement of Mitochondrial Membrane Potential (ΔΨm)

The ΔΨm value was measured with fluorescent carbocyanine dye JC1 that enters the mitochondria and exhibits red fluorescence (FL) in a ΔΨm dependent manner [13]. JC1 retained in the cytosol displays green FL. The cells were stained with JC1 (2 *μ*M) in culture media for 30 min and washed with cold PBS 5 times, and the fluorescence was recorded with a Synergy H4 Hybrid Multi-Mode Microplate Reader (BioTek), set at 488 nm excitation and 530 nm (green) and 585 nm (red) emission [34]. The ratio of red FL against green FL reflects ΔΨm of the mitochondria [13, 34].

### 2.6. Glycolytic Activity

The glycolytic activity was analyzed with Glycolysis Cell-Based Assay Kits (Cayman Chemical) according to the manufacturer's instructions. Briefly, the cells were maintained or treated as described above until the day before measurement, when the cells were switched to regular media with no FBS for 20 hours in a 5% CO_2_ incubator at 37°C, and the media were taken to measure glycolytic activity.

### 2.7. Immunohistochemistry Analysis of Human Breast Tissues

Archive paraffin blocks of breast cancer tissues and adjacent normal tissues from human breasts were used for immunohistochemical analysis using a rabbit polyclonal Drp1 antibody (NB110-55237, Novus Biologicals) and a mouse monoclonal anti-BNIP3 antibody (ab10433, Abcam) by following the manufacturers' instruction. Hematoxylin was used for counterstaining. Slides were examined with a Nikon Eclipse 80i microscope.

### 2.8. Bioinformatics Study

The gene expression patterns of DNM1L (encoding Drp1) in breast cancer tissues were studied with The Cancer Genome Atlas (TCGA) database at Insilicom (http://insilicom.com/). Insilicom integrates large volume and heterogeneous oncology data deposited at TCGA. We conducted the queries targeting DNM1L in normal breast tissues and breast cancers (BRCA), which led to data sets (mRNA microarray) containing 529 breast cancer entities and 61 normal breast tissue entities. The array data in log⁡2 expression values in breast cancers were converted into 2^*N*^ format and normalized against normal breast tissues for mean fold changes and statistics analysis (ANOVA).

### 2.9. Western Blot

The cells were harvested and washed with ice-cold PBS (phosphate buffered saline, Caisson Labs), followed by lysis with Bullet Blender® (Next Advance, Inc.) in PLC lysis buffer [13, 35]: 30 mM Hepes, pH 7.5, 150 mM NaCl, 10% glycerol, 1% Triton X-100, 1.5 mM MgCl_2_, 1 mM EGTA, 10 mM NaPPi, 100 mM NaF, 1 mM Na_3_VO_4_ supplemented with protease inhibitor cocktail (Roche), 1 mM PMSF, 10 *μ*M TSA (Trichostatin A, Selleckchem), and 5 mM nicotinamide (Alfa Aesar). Total protein concentrations of the cell lysates were determined using the DC protein assay (Bio-Rad). Western blot and image analysis were conducted as described previously [13]. Antibody catalog numbers and vendors are as follows: C1 (A31857) and C3 (A21362) from Invitrogen; BNIP3 (ab10433) from Abcam; *β*-actin (MA5-15739), GAPDH (MA5-15738), and LC3 (PA1-16931) from Pierce; Mfn1 (sc-50330), Mfn2 (sc-50331), and Tfam (sc-28200) from Santa Cruz; PGC1*α* (Ab3242) from Millipore; VDAC (4661s) and Drp1 (8570) from Cell Signaling Technology; Beclin-1 3738s and Beclin-1 MABN16 from Cell Signaling Technology and Millipore, respectively; NRF1 (LS-B43) from LifeSpan BioSciences.

### 2.10. Statistical Analyses

All results are expressed as means ± SEM and are analyzed by analysis of variance (ANOVA) to determine *p* values; *p* < 0.05 was considered statistically significant.

## 3. Results

### 3.1. Transcript and Protein Levels of Drp1 Were Upregulated in Breast Cancer

**Figure 1:**
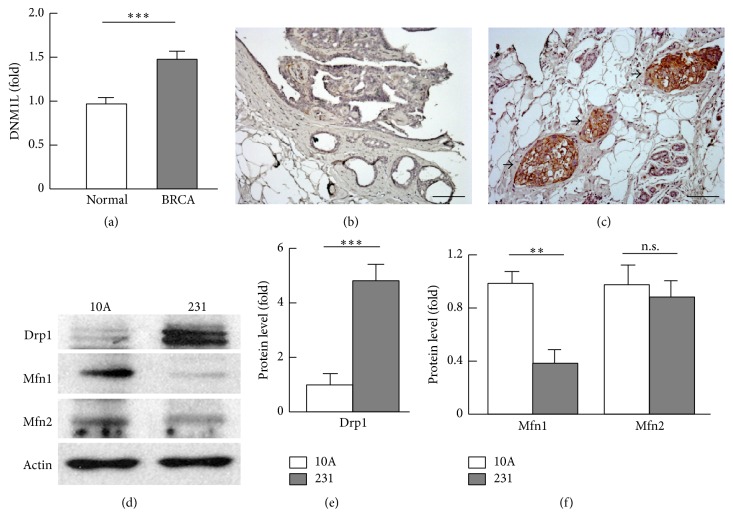
Analysis of mitochondrial dynamics regulators. (a) Gene expression of DNM1L (encoding the mitophagy activator Drp1) in normal breast tissues (*n* = 61) and breast cancer (BRCA, *n* = 529) tissues. The data were extracted from TCGA (The Cancer Genome Atlas) database at Insilicom (http://insilicom.com/). (b)-(c) Immunohistochemistry analysis of Drp1 in normal breast (b) and invasive carcinoma (c) tissues. Scale bar: 100 *μ*m; arrows indicate massively positive staining of Drp1 in the breast cancer tissues. (d) Western blot analysis of mitochondrial fission (Drp1) and fusion (Mfn1 and Mfn2) proteins in MCF-10A (abbreviated as “10A”) and MDA-MB-231 (abbreviated as “231”) cells. (e)-(f) Densitometric analysis of western blot images as shown in (d) (*n* = 3–5). ^*∗∗*^ *p* < 0.01; ^*∗∗∗*^ *p* < 0.001; n.s.: not significant.

Activation or overexpression of Drp1 protein has been implicated in oncogenic pathways, tumor growth, and metastatic process [14–18]. To determine whether gene expression of Drp1 is dysregulated, we analyzed the transcript level of DNM1L (encoding Drp1) in 529 human breast cancer tissues and 61 normal breast tissue entities, which showed significant upregulation of DNM1L in breast cancer tissues (Figure 1(a)). Immunohistochemistry (IHC) analysis of normal breast tissues and invasive carcinomas revealed strong staining of Drp1 in the cancer tissues but not in normal breast tissues (Figures 1(b) and 1(c)). Western blot analysis of Drp1 protein in breast cancer MDA-MB-231 cells indicated 5.1-fold (*p* < 0.001) elevation in comparison with nontumorigenic human breast MCF10A cells (Figures 1(d) and 1(e)). Interestingly, mitochondrial fusion protein Mfn1 was downregulated by 60% (*p* < 0.01), which was associated with a significant reduction of Mfn1 gene expression; however, Mfn2 mRNA and protein were unchanged (Figures 1(d) and 1(f) and supplemental Figure 1 in Supplementary Material available online at http://dx.doi.org/10.1155/2016/4085727). These results suggest that breast cancer adopts selective mitochondrial dynamics which favor fission over fusion by upregulating Drp1 but downregulating Mfn1.

### 3.2. Mitochondrial Content and Oxidative Capacity Were Reduced in Breast Cancer Cells

**Figure 2 fig2:**
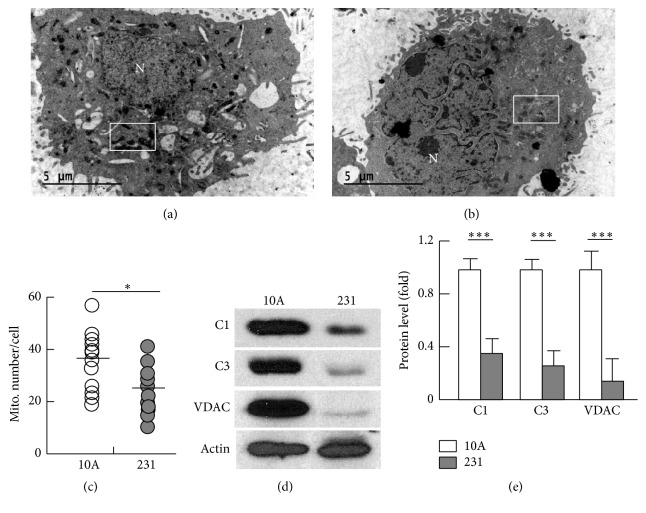
Analysis of mitochondrial content. (a)-(b) Electron microscopic view of ultrathin sections of MCF-10A (a) and MDA-MB-231 cells (b). See supplemental Figure 2 for images at a higher magnification. The letter “N” denotes nucleus, and the rectangles indicate mitochondria that show high (distinctly darker) (a) and low (b) electron density. (c) Count of mitochondrial numbers using electron microscopy, with median values indicated; *n* = 12. (d) Western blot analysis of mitochondrial proteins complex I (C1), complex III (C3), and VDAC. (d) Densitometric analysis of western blot images as shown in (d) (*n* = 3–5). ^*∗*^ *p* < 0.05 and ^*∗∗∗*^ *p* < 0.001.

To determine how dysregulated mitochondrial dynamics might affect mitochondrial content and quality in breast cancer cells, we employed electron microscopy to study the ultrathin sections of the cells. As shown in Figures 2(a) and 2(b) and supplemental Figure 2, the mitochondria in MDA-MB-231 cells had lower electron density in the matrix than in MCF-10A cells, indicative of metabolically less active or unhealthy mitochondria [13, 36, 37]. In line with upregulated Drp1 and downregulated Mfn1 (Figure 1), the mitochondria were smaller or more fragmented in MDA-MB-231 cancer cells (supplemental Figure 2). Moreover, the mitochondrial number in MDA-MB-231 cells was reduced by 34% when compared with MCF-10A cells (a median value of 25.5 versus 38.5 mitochondria/cell, resp., *p* < 0.05) (Figures 2(a)–2(c)). The reduced mitochondrial content was further validated by a significant decrease in the protein level of VDAC (82%, *p* < 0.001), a biomarker of mitochondrial content [38] (Figures 2(d) and 2(e)). In addition, the expression of ETC components, complexes I (C1) and III (C3), was downregulated by 67% (*p* < 0.001) and 75% (*p* < 0.001), respectively (Figures 2(d) and 2(e)). Following an established protocol, we measured the oxygen consumption rate (OCR), mitochondrial ATP turnover, respiration control ratio, coupling efficiency, max respiration rate, and spare respiration capacity [33], which revealed a significantly compromised mitochondrial oxidative capacity in MDA-MB-231 cells (supplemental Figure 3, A–E). Consistent with these changes, mitochondrial coupling efficiency (CE) and membrane potential (ΔΨm) were reduced in MDA-MB-231 cells (supplemental Figure 3, F-G).

### 3.3. Mitochondrial Biogenesis Was Enhanced in Breast Cancer Cells

**Figure 3 fig3:**
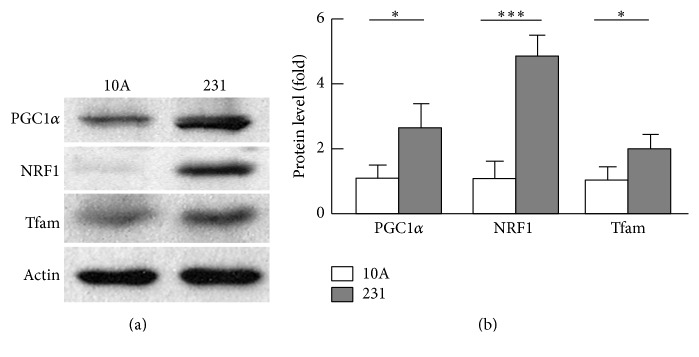
Analysis of mitochondrial biogenesis regulators. (a) Western blot analysis of the regulators of mitochondrial biogenesis (PGC1*α*, NRF1, and Tfam). (b) Densitometric analysis of western blot images as shown in (a) (*n* = 3–5). ^*∗*^ *p* < 0.05; ^*∗∗∗*^ *p* < 0.001.

The reduced mitochondrial number prompted us to examine whether mitochondrial biogenesis was downregulated. Unexpectedly, the key activators of mitochondrial biogenesis, PGC1*α* and its downstream targets NRF1 and Tfam [39–41], were upregulated by 2.9-fold (*p* < 0.05), 4.8-fold (*p* < 0.001), and 2.1-fold (*p* < 0.05), respectively, in MDA-MB-231 cells when compared with MCF-10A cells (Figures 3(a) and 3(b)). The paradoxical reduction of mitochondrial number versus increased mitochondrial biogenesis and fission suggests that breast cancer cells may adopt an overactive process of mitochondrial turnover.

### 3.4. Mitochondrial Autophagy Was Upregulated in Breast Cancer

**Figure 4:**
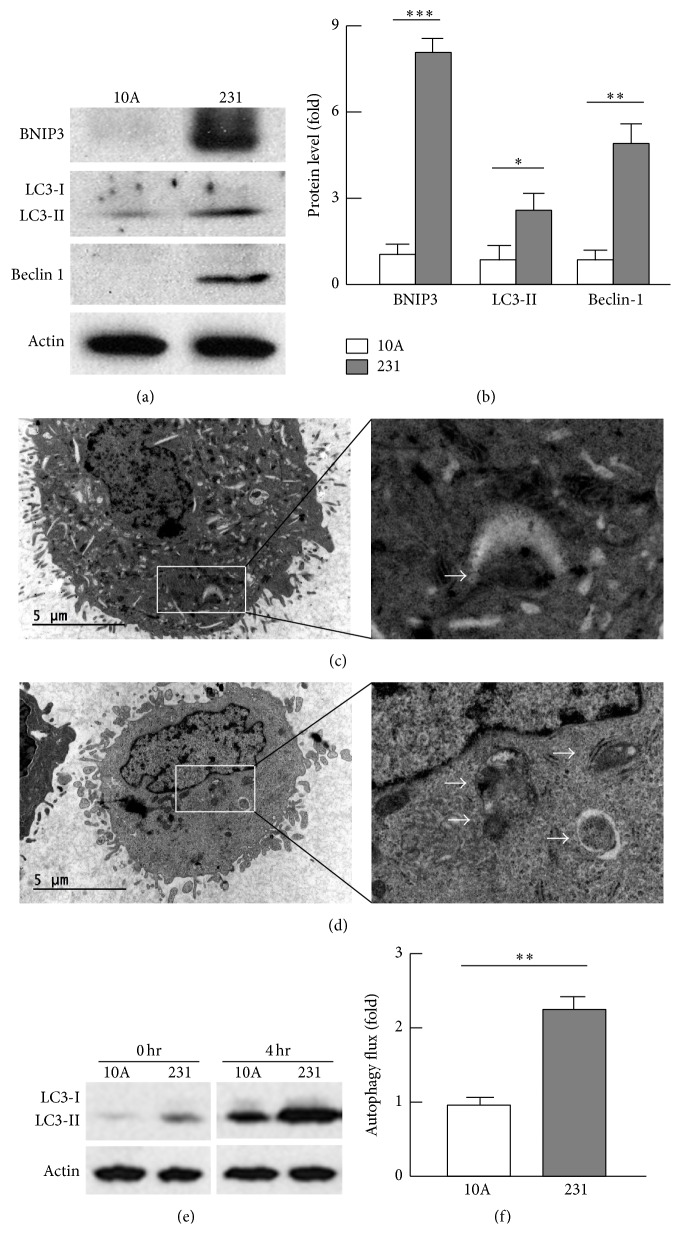
Analysis of mitochondrial autophagy. (a)-(b) Analysis of the mitophagy biomarker (BNIP3) and autophagosome regulators (beclin-1 and LC3-II) by western blot (a) and densitometry (b). (c)-(d) Electron microscopic imaging revealed higher population of autophagic vacuoles engulfing mitochondria (indicated by arrows) in MDA-MB-231 (d) than in MCF-10A (c) cells. See supplemental Figure 2 for images at a higher magnification. (e)-(f) Measurement of autophagic flux by western blot (e) and densitometry (f). Autophagic flux was calculated as the difference between LC3-II levels in the absence (0 hr) and presence (4 hr) of autolysosome inhibitors bafilomycin A1 (0.1 *μ*M) and leupeptin (10 *μ*g/mL); *n* = 3–5; ^*∗*^ *p* < 0.05, ^*∗∗*^ *p* < 0.01, and ^*∗∗∗*^ *p* < 0.001.

Mitochondrial autophagy (or mitophagy) plays an important role in mitochondrial turnover and quality control [42]. To test whether mitophagy is involved in mitochondrial changes in cancer cells, we analyzed the mitophagy marker BNIP3 [30, 43] and found that its protein level was 7.9-fold elevated (*p* < 0.001) in MDA-MB-231 cells when it was compared with that in MCF-10A cells (Figures 4(a) and 4(b)). By immunohistochemistry analysis, we observed dense staining of BNIP3 in invasive carcinomas, which was absent from normal breast tissues (supplemental Figure 4), suggestive of upregulated mitochondrial autophagy in breast cancer. Indeed, we observed a larger population of autophagic vacuoles that engulfed mitochondria in MDA-MB-231 cells than in MCF-10A cells by electron microscopic study (Figures 4(c) and 4(d) and supplemental Figure 2). Accordingly, the protein levels of beclin-1 and LC3-II that contribute to autophagosome formation and maturation [30, 44, 45] were 5.4-fold (*p* < 0.05) and 2.9-fold (*p* < 0.01) increased, respectively (Figures 4(a) and 4(b)). Measurement of LC3-II level in the absence and presence of bafilomycin A1 and leupeptin (autolysosome inhibitors) [27–30] indicated that MDA-MB-231 cells had an autophagic flux 2.3 times (*p* < 0.01) higher than MCF-10A cells (Figures 4(e) and 4(f)). Taken together, these results demonstrate an increased removal of mitochondria via mitophagy in breast cancer cells, which may account for the reduced mitochondrial content.

### 3.5. Inhibition of Drp1 Suppressed Mitochondrial Autophagy, Metabolic Reprograming, and Cancer Cell Viability

**Figure 5:**
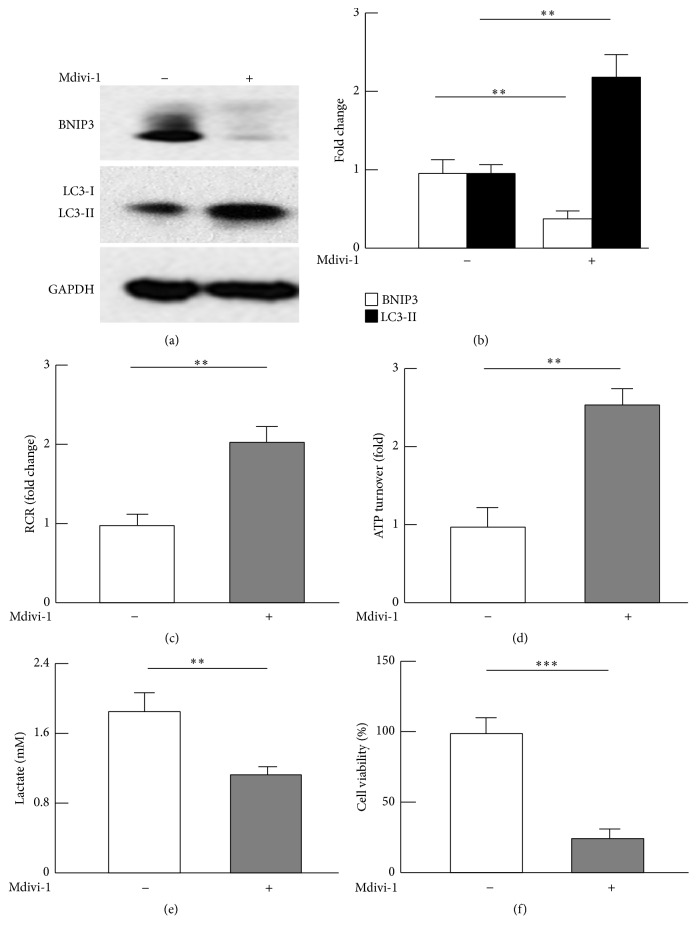
Effects of Drp1 inhibitor Mdivi-1 on mitochondrial autophagy, metabolism, and viability of MDA-MB-231 cancer cells. (a)-(b) Mdivi-1 suppressed mitophagy marker BNIP3 and caused accumulation of LC3-II, indicative of reduced autophagic flux. MDA-MB-231 cells were cultured and treated with Mdivi-1 as described in the Materials and Methods, and the protein levels were analyzed by western blot (a) and densitometric (b) analysis; *n* = 3–5. (c)-(d) Mdivi-1 promoted mitochondrial oxidative capacity, as evaluated by respiratory control ratios (RCR) and ATP turnover. (e) Mdivi-1 suppressed glycolysis (*n* = 3). (f) Mdivi-1 reduced breast cancer cell viability (*n* = 5–7). ^*∗∗*^ *p* < 0.01 and ^*∗∗∗*^ *p* < 0.001.

Previous studies suggest that mitophagy is accompanied with enhanced mitochondrial fission [46, 47], which prompted us to examine whether Drp1 upregulation accounted for the augmented mitophagy in breast cancer cells. Using an established Drp1 antagonist (Mdivi-1) [21–24], we treated breast cancer MDA-MB-231 cells and measured the protein levels of BNIP3 and LC3-II. Intriguingly, Mdivi-1 dramatically suppressed mitophagy marker BNIP3, concomitant with accumulation of LC3-II that indicates reduced autophagic flux (Figures 5(a) and 5(b)) [27–30]. However, Mdivi-1 did not affect beclin-1, suggesting that Mdivi-1 or Drp1 may mediate mitochondrial autophagy downstream of beclin-1 (supplemental Figure 5). In addition, the effect of Mdivi-1 on mitochondrial biogenesis marker was marginal (supplemental Figure 5). Of significance, Mdivi-1 increased mitochondrial respiratory control ratio (RCR) and ATP turnover oxidative capacity by 2.0–2.5 times (*p* < 0.01; Figures 5(c) and 5(d)) and attenuated lactate production or activity of glycolysis (43%, *p* < 0.01; Figure 5(e)). These results strongly suggest that Mdivi-1 leads to a reversal of the metabolic switch known as Warburg effect in cancer metabolism [4–6]. Moreover, Mdivi-1 treatment led to a substantial decrease in cancer cell viability (78%, *p* < 0.001) (Figure 5(f)). Therefore, Drp1-induced mitophagy may account largely for the metabolic reprograming and cancer cell survival.

## 4. Discussion

Overactivation of mitochondrial fission was recently shown to promote tumorigenesis and cancer invasion [14–18]. An important question to be addressed is how mitochondrial quality control is regulated by overactive mitochondrial division. We employed bioinformatics approach analyzing 529 human breast cancer entities from The Cancer Genome Atlas (TCGA) database, and the results were validated by experimental biology in human breast cancer tissues and cell cultures. Our data suggest that breast cancer adopts a coordinated upregulation of mitophagy and mitochondrial biogenesis during the selective dynamics favoring mitochondrial fission (Figures 1 [Fig fig2] [Fig fig3]–4). Moreover, we showed that upregulation of Drp1 directly contributed to augmented mitophagy, which accounted at least in part for the increased mitochondrial turnover and metabolic reprograming in breast cancer cells (Figures 4 and 5). Activation of Drp1 to promote mitochondrial fission has been well established in general. However, the evidence for the physiological role of Drp1-induced mitophagy has just started to emerge, including maintenance of functional *β*-cells and cardio-/neuroprotections [22, 46–48]. To our knowledge, this is the first study investigating the triad of mitochondrial biogenesis, dynamics, and autophagy in mitochondrial quality control with regard to cancer metabolism.

It is noteworthy that mitochondrial biogenesis pathway via PGC1*α* is activated in breast cancer cells. Because a basal mitochondrial function is required for cancer cell survival and tumor growth (e.g., generating chemical building blocks for biosynthesis) [4, 5], upregulation of PGC1*α*-mediated mitochondrial biogenesis may play a critical role in meeting such a requirement, particularly in the case of augmented mitochondrial turnover (mitophagy) induced by Drp1. Indeed, recent studies suggested that expression of ectopic PGC1*α* promoted tumor growth in an ErbB2/Neu-induced breast cancer, and elevation of PGC1*α* expression was correlated with a lower patient survival rate [49, 50]. These findings highlight the importance of considering both mitochondrial biogenesis and mitophagy for effective cancer treatment. Indeed, targeting mitophagy or BNIP3 pathway alone was paradoxically shown to promote or suppress tumorigenesis [51–56]. This complexity and controversy may arise from the coordinated increase of mitochondrial biogenesis and mitophagy. Thus, it is of critical importance for future studies to test the effectiveness of simultaneously targeting mitophagy and mitochondrial biogenesis for breast cancer treatment.

## Supplementary Material

In line with dysregulated mitochondrial dynamics, Mfn1 transcript increased significantly in breast cancer and mitochondrial oxidative metabolism was impaired in MDA-MB-231 cancer cells. Mitochondria were smaller and fewer, with lower electron density in breast cancer cells than in non-tumorigenic human breast MCF10A cells. The reduced mitochondrial content was associated with upregulated mitophagy marker BNIP3 in breast cancer. Inhibition of mitochondrial fission or Drp1 with Mdivi-1 had marginal effect on mitochondrial biogenesis marker PGC1a.

## Abbreviations

ATP: Adenosine triphosphate
BECN1: The gene encoding the autophagy marker beclin BNIP3, BCL2/adenovirus E1B 19 kDa interacting protein 3
C1: Mitochondrial complex I
C3: Mitochondrial complex III
CE: Coupling efficiency
ΔΨm: Membrane potential
DNM1L: The gene encoding Drp1
Drp1: Dynamin related protein-1
ER: Endocrine receptor (estrogen or progesterone receptor)
ETC: Electron transport chain
PGC1*α*: Peroxisome proliferator activated receptor gamma coactivator 1 alpha
IHC: Immunohistochemistry
LC3: Microtubule-associated protein-1 light chain 3 alpha
LC3-II: LC3-phosphatidylethanolamine conjugate
Mdivi-1: 3-(2,4-Dichloro-5-methoxyphenyl)-2,3-dihydro-2-thioxo-4(1H)-quinazolinone, 3-(2,4-dichloro-5-methoxyphenyl)-2-sulfanyl-4(3H)-quinazolinone
Mfn1: Mitofusin 1
Mfn2: Mitofusin 2
NRF1: Nuclear respiratory factor-1
OCR: Oxygen consumption rate
OXPHOS: Oxidative phosphorylation
RCR: Respiratory control ratio
SRC: Spare respiration capacity
Tfam: Mitochondrial transcription factor A
VDAC: Voltage-dependent anion channel.
