# Supplementary material for: Coordinated Upregulation of Mitochondrial Biogenesis and Autophagy in Breast Cancer Cells: The Role of Dynamin Related Protein-1 and Implication for Breast Cancer Treatment
Source: Oxid Med Cell Longev. 2016 Sep 26;2016:4085727. doi: 10.1155/2016/4085727 (PMC5056295; doi:10.1155/2016/4085727)
Supplement: Supplementary file 1 — In line with dysregulated mitochondrial dynamics, Mfn1 transcript increased significantly in breast cancer and mitochondrial oxidative metabolism was impaired in MDA-MB-231 cancer cells. Mitochondria were smaller and fewer, with lower electron density in breast cancer cells than in non-tumorigenic human breast MCF10A cells. The reduced mitochondrial content was associated with upregulated mitophagy marker BNIP3 in breast cancer. Inhibition of mitochondrial fission or Drp1 with Mdivi-1 had marginal effect on mitochondrial biogenesis marker PGC1a. [file 4085727.f1.docx]

**Supplemental Figure 1**. Gene expression of MFN1 and MFN2 in normal breast tissues (n=61) and breast cancer (BRCA, n=529) tissues. The data were extracted from TCGA (The Cancer Genome Atlas) database at Insilicom (http://insilicom.com/). ***, *p*<0.001; n.s., not significant.

**Supplemental Figure 2.** High-magnification imaging of ultrathin sections of MCF-10A (panel A) and MDA-MB-231 (panel B) cells under electron microscopy. MCF-10A cells showed bigger and less fragmented mitochondria with higher electron density (i.e., distinctly darker) than MB-231 cells. In addition, MB-231 cells had greater population of autophagic vacuoles engulfing mitochondria (indicated by arrows) in MDA-MB-231 than in MCF-10A cells.

**Supplemental Figure 3.** Measurement of oxygen consumption rate and mitochondrial oxidative capacity. MCF-10A (abbreviated as “10A”) and breast cancer MDA-MB-231 (abbreviated as “231”) cells were cultured and underwent analysis of oxygen consumption rate (OCR, panel A), mitochondrial ATP turnover (panel B), respiratory control ratio (RCR, panel C), max respiration rate (panel D), spare respiration capacity (SRC, panel E), coupling efficiency (CE, panel F), and membrane potential (Δψm, panel G), as described in “Materials and Methods”. Results were expressed as means ± SD (n =3). *, *p*<0.05, and **, *p*<0.01.

**Supplemental Figure 4**. Immunohistochemistry analysis of BNIP3 in normal breast (A) and invasive carcinoma (B) tissues. Scale bar: 100μm; arrows indicate massively positive staining of BNIP3 in the breast cancer tissues.

**Supplemental Figure 5**. Effects of Drp1 inhibitor Mdivi-1 on beclin 1 and PGC1α. MDA-MB-231 cancer cells were cultured and treated with Mdivi-1 as described in Materials and Methods. The protein levels of beclin 1 and PGC1α were measured by western blot analysis. Results were expressed as means ± SD (n =3).
